# Use, Safety Assessment, and Implementation of Two Point-of-Care Tests for COVID-19 Testing

**DOI:** 10.1093/ajcp/aqab081

**Published:** 2021-07-24

**Authors:** Megan Hahn, Aaron Olsen, Kindra Stokes, Randal C Fowler, Rui Gu, Shellanne Semple-Lytch, Andrea DeVito, Philip Kurpiel, Scott Hughes, Jennifer L Rakeman

**Affiliations:** 1 Department of Health and Mental Hygiene, New York City Public Health Laboratory, New York, NY, USA; 2 Centers for Disease Control and Prevention, Atlanta, GA, USA

**Keywords:** SARS-CoV-2, Abbott ID NOW COVID-19, Quidel Sofia 2 SARS, Usability, Point-of-care implementation

## Abstract

**Objectives:**

The Abbot ID NOW COVID-19 assay and Quidel Sofia 2 SARS Antigen FIA are point-of-care assays that offer rapid testing for severe acute respiratory syndrome coronavirus 2 viral RNA and nucleocapsid protein, respectively. Given the utility of these devices in the field, we investigated the feasibility and safety of using the ID NOW and Sofia assays in the public health response to the coronavirus disease 2019 pandemic and in future public health emergencies.

**Methods:**

A combination of utilization and contamination testing in addition to a review of instrument workflows was conducted.

**Results:**

Utilization testing demonstrated that both tests are intuitive, associated with high user test success (85%) in our study, and could be implemented by staff after minimal training. Contamination tests revealed potential biosafety concerns due to the open design of the ID NOW instrument and the transfer mechanisms with the Sofia. When comparing the workflow of the ID NOW and the Sofia, we found that the ID NOW was more user-friendly and that the transfer technology reduces the chance of contamination.

**Conclusions:**

The ID NOW, Sofia, and other emerging point-of-care tests should be used only after careful consideration of testing workflow, biosafety risk mitigations, and appropriate staff training.

**Key Points**

- The Quidel Sofia SARS 2 Antigen FIA and Abbott ID NOW COVID-19 assay are intuitive and could be implemented by staff after minimal user training.
- The Quidel Sofia SARS 2 Antigen FIA and Abbott ID NOW COVID-19 assay and other emerging point-of-care tests should be used only after careful consideration of testing workflow.
- Through workflow evaluation, we provide a toolkit that can be used to implement point-of-care tests in a variety of settings.

The availability and utility of authorized Clinical Laboratory Improvement Amendments waived tests (https://www.cdc.gov/labquality/waived-tests.html) for nonlaboratory field applications have revolutionized the way that we address testing during disease outbreaks, epidemics, and pandemics. These point-of-care tests (POCTs) require relatively little training and laboratory experience and allow for rapid time to results. If an instrument is required for the test, it is generally small and portable. These features allow health care providers to (1) identify individuals that may further spread disease, (2) reduce the risk of transmission in congregate settings, (3) make rapid clinical decisions, and (4) run or care for the devices with a minimal need for training. As a result, POCTs enable testing at remote care centers and field locations and in some instances may provide a lower overall cost per test than laboratory-based tests and facilitate greater frequency of testing.^1-3^ While POCTs have been in use for the past two decades to diagnose diseases, including streptococcal throat infections and influenza, the coronavirus disease 2019 (COVID-19) pandemic has highlighted their ability to increase general testing availability in emergency situations.^4-7^ Testing demand and impact of the pandemic on production and availability of supplies for testing for severe acute respiratory syndrome coronavirus 2 (SARS-CoV-2) has resulted in shortages of swabs, viral transport media, and RNA extraction kits.^4-7^ Due to limited reagents and collection supplies, laboratory testing capacity is often not able to meet testing demand at the beginning of a pandemic response, as was observed with SARS-CoV-2. As pandemic response progresses and laboratory testing expands, clinics, schools, and community testing sites then may not have the capacity to meet surveillance demands. This combination of limited clinical testing capacity and abundance of cases has prompted international public health initiatives to ramp up COVID-19 field testing and, through this, the use of POCTs.^8^

We designed a study to evaluate the benefits and drawbacks of two instrument-based POCTs—the Abbott ID NOW COVID-19 test (ID NOW), which is an RNA detection test for SARS-CoV-2, and the Sofia 2 SARS Antigen FIA test (Sofia)—in nonlaboratory settings staffed by assay users with minimal experience using the instruments **Table 1**. Overall, we found that while both instruments have potential for use in nonlaboratory settings, staff will require proper training and practice to avoid potential contamination and unintended spread of infectious material.

**Table 1:** Instrument Comparison

Characteristic	ID NOW	Sofia
Type of assay	Polymerase chain reaction	Antigen FIA
Turnaround time, min	20	15
Sample type	Swab	Swab
Specimen type	Respiratory specimens; nasopharyngeal or nares swabs	Respiratory specimens; nasopharyngeal or nares swabs
No. of samples that can be processed at a time	1	1

## Materials and Methods

### Testing the ID NOW Limit of Detection

The limit of detection (LOD) was determined for the ID NOW to calculate the amount of standard reference material appropriate for user testing. Dilutions were made by spiking directly into the sample receiver so that the reaction cartridge contained 8,000 genome copies down to 250 copies per reaction (Exact Diagnostics, cat. COVO19). The number of genome copies per milliliter was calculated taking into account the 2.5 mL of lysis buffer in the sample receiver plus 0.2 mL of spiked material. The final LOD was determined as the lowest concentration that could be detected 95% or more of the time in at least 20 replicates.

### Testing the Clinical Performance of the Sofia

The clinical performance for the Sofia 2 SARS Antigen FIA was determined. Clinical specimens delivered to the New York City Public Health Laboratory (NYC PHL) with cycle threshold (C_T_) values obtained using the New York SARS-CoV-2 real-time reverse transcription (RT)–polymerase chain reaction (PCR) diagnostic test^9^ ranging from 17 to 36.2 were used. Specimens were categorized with C_T_ values under 20 (low), 20 to 25 (medium), 25.1 to 30 (medium-high), and 30.1 and greater (high). Testing determined sensitivity and specificity in the same manner as the manufacturer.^10^ LOD was not determined because appropriate facilities and reagents were not available to complete this for an antigen-based test.

### Recruitment of Users

Participants in the study were recruited from NYC PHL staff. Five users who ranged in their laboratory experience were selected to simulate differences in testing proficiency that may be observed at different testing and point-of-care sites. One user had 10+ years of laboratory experience but did not perform work in the laboratory regularly. Another user had 5 years of laboratory experience and worked daily shifts in a laboratory. The remaining three users had minimal or no laboratory experience **Table 2**. None of the users had any experience with either the ID NOW or the Sofia test. Nontechnical staff were given blinded, spiked samples containing either SARS-CoV-2 reference material or sterile saline and assessed for their ability to accurately perform testing. Glo Germ (Glo Germ Company, cat. GGG8O) was used to visually track the potential for sample contamination of work surfaces and users’ personal protective equipment (PPE) during and following testing. Users were also interviewed and shadowed to identify potential workflow challenges and POCT system preference.

**Table 2:** Laboratory Experience of Users^a^

System User	Status	Technical Laboratory Experience, y
1	Active laboratory staff	5
2	Nonlaboratory staff	0
3	Nonactive laboratory staff	>10
4	Nonlaboratory staff	0
5	Nonlaboratory staff	0

^a^Laboratory staff are those with previous training (research, clinical, or medical) and experience (years) working in a laboratory setting. The term *active* refers to a staff member who is currently working in the laboratory on a regular basis. *Nonactive* refers to a staff member who has laboratory experience but whose current role has them primarily out of the laboratory. Nonlaboratory staff are those who have minimal to no previous laboratory training or experience working in a laboratory setting.

### Blinded Study of Five Users Using Negative Control and Spiked Reference Material

The five newly trained assay users performed blinded tests on the ID NOW on the open benchtop using three positive samples (noninfectious material spiked in saline at 2×, 5×, 10× LOD) and two negative samples (matrix only). Users were first supplied with the ID NOW COVID-19 product insert to read, and proper laboratory technique was demonstrated by experienced staff. Environmental samples were collected by swabbing the instruments and the work surfaces surrounding the instruments prior to and between each consecutive test and following the completion of testing by each user.

Testing with the Sofia was conducted in the same manner as with the ID NOW, with the following modifications. To allow for open benchtop testing, protect the users, and standardize all tests, samples spiked with 1 µg of recombinant nucleocapsid protein were used to create contrived “positive” samples for the Sofia testing (ExonBio, cat. 19Cov-N150). The concentration of protein in simulated samples was tested by experienced staff on the Sofia prior to testing to confirm that positivity was obtained.

### ID NOW and Sofia Workflow Assessment

Workflow challenges associated with the use of ID NOW and Sofia were assessed by observing the five newly trained assay users during testing. An evaluation checklist (Supplementary Box 1; all supplemental materials can be found at *American Journal of Clinical Pathology* online) was completed for each user (1-5), as the evaluators assessed the following: (1) knowledge of the testing procedures and (2) sample handling techniques. Glo Germ luminescent detection system was used as a mock sample to assess sample handling. Following the manufacturer’s instructions, users processed mock samples on an open benchtop. LED UV flashlights and photos were used to visually capture potential contamination zones on the instruments, disposable consumables, surrounding workspaces, and the users’ PPE. Following each user test, the instruments and workspaces were decontaminated, and decontamination was confirmed by repeating the UV light luminescent detection.

To confirm that environmental contamination on the work surface could be detected during testing should a splash or spill occur, 100 µL of reference material from the ID NOW and the Sofia was applied to a sterilized bench and allowed to dry. The dried surface was then swabbed and eluted into viral transport media and tested using the Cepheid Xpert Xpress SARS-CoV-2 test per the manufacturer’s instructions for use.^11^

## Results

### Analytical Performance and Utilization Testing

The LOD was determined to be 556 copies/mL with 95% detection achieved in 20 sample replicates at that concentration **Table 3**. Testing of clinical specimens on the Sofia revealed 92% were correctly identified as positive and 60% were correctly identified as negative with an overall agreement of 80% using New York SARS-CoV-2 RT-PCR diagnostic panel as the comparator assay **Table 4**.^9^ In-house testing showed that the Sofia was estimated to have an 80% sensitivity and specificity for testing with a single user.

**Table 3:** Abbott ID NOW Limit of Detection Determined Using Contrived Samples^a^

No. of Genome Copies per Reaction	No. of Genome Copies per mL	Detected/Not Detected
8,000	2,963	Detected
4,000	1,481	Detected
2,000	741	Detected
1,500	556	Detected
1,000	370	Not detected
500	185	Not detected
250	93	Not detected

^a^A series of dilutions was created in triplicate and tested using the ID NOW COVID-19 assay.

**Table 4:** Sofia 2 SARS Antigen FIA Clinical Performance Determined Using Clinical Specimens^a^

	New York State SARS-CoV-2 RT-PCR, No.			
Quidel Sofia Result	Positive	Negative	Total No.	% Correct
Positive	36	3	39	92
Negative	9	12	21	60
Total	45	15	60	

^a^Nasal pharyngeal specimens of varying cycle threshold values determined using the New York SARS-CoV-2 real-time reverse transcription–polymerase chain reaction (RT-PCR) diagnostic panel were selected and tested using the Sofia assay to determine predictive values (the percentage of specimens tested that the correct result was obtained for).

Five newly trained assay users tested a panel of three spiked samples and two negative controls in a blinded fashion using the ID NOW following the order indicated in **Table 5**. The low positive sample (2× LOD) resulted in false negatives for 40% (two of five) of users. In addition, one invalid result was due to a sample loading error, which was corrected after the sample was reloaded and tested again. All users obtained the expected result for the medium (5× LOD) and high (10× LOD) samples, as was also true for all negative control samples.

**Table 5:** Results From Blinded Samples Tested by New Users Performed on the ID NOW COVID-19 Test or Sofia 2 SARS Antigen FIA^a^

ID NOW Panel					
User	Sample 1, Negative Control	Sample 2, Spike 5× LOD	Sample 3, Negative Control	Sample 4, Spike 2× LOD	Sample 5, Spike 10× LOD
1	Pass	Pass	Pass	Fail	Pass
2	Pass	Pass	Pass	Invalid/pass^b^	Pass
3	Pass	Pass	Pass	Pass	Pass
4	Pass	Pass	Pass	Fail	Pass
5	Pass	Pass	Pass	Pass	Pass
Sofia Panel					
User	Sample A, Negative Control	Sample B, Spike 5× LOD	Sample C, Negative Control	Sample D, Spike 2× LOD	Sample E, Spike 10× LOD
1	Pass	Pass	Pass	Pass	Pass
2	Pass	Fail	Pass	Fail	Fail
3	Pass	Pass	Pass	Pass	Pass
4	Pass	Pass	Pass	Pass	Pass
5	Pass	Pass	Pass	Pass	Pass

LOD, limit of detection.

^a^Newly trained assay users were given blinded samples containing saline spiked with viral transcript for the ID NOW, nucleocapsid protein for the Sofia, or negative control. Testing occurred with each of the five users performing five consecutive tests. For the ID NOW testing, two samples spiked at 2× LOD failed to be detected. All others passed, including negative controls. For the Sofia testing, we observed that four of five users correctly identified all samples, and the fifth user was unable to successfully identify positive samples.

^b^The first result was invalid due to a loading error. The sample passed after it was reloaded and run again.

Similarly, the same five assay users tested a panel on the Sofia, which consisted of three spiked samples and two negative controls. Antigen was not detected in 20% (3/15) of positive samples. All false negatives were tested by a single user while the remaining users correctly identified all positive specimens. All users failed to detect antigen in negative samples.

### ID NOW COVID-19 Workflow Assessment

**Figure 1:**
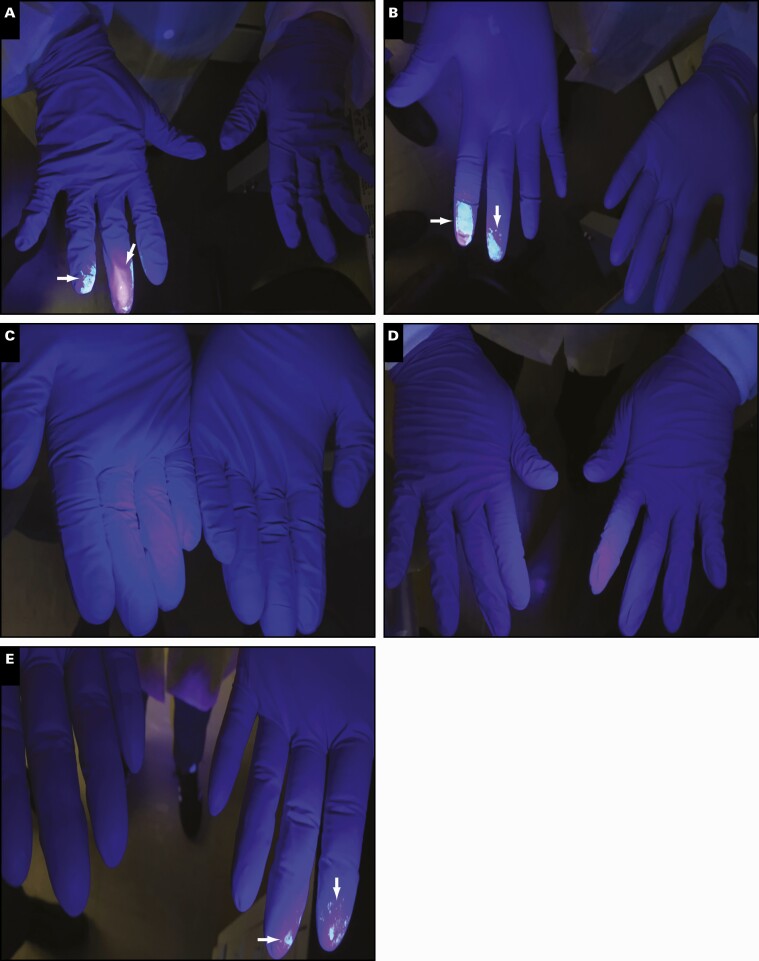
ID NOW visualization of Glo Germ cross-contamination following mock sample handling. Representative of users’ gloves after processing mock Glo Germ sample on the ID NOW. Contamination zones denoted by arrows. **A**, User 1, laboratory staff. **B**, User 2, nonlaboratory staff. **C**, User 3, laboratory staff. **D**, User 4, nonlaboratory staff. **E**, User 5, laboratory staff.

**Figure 2:**
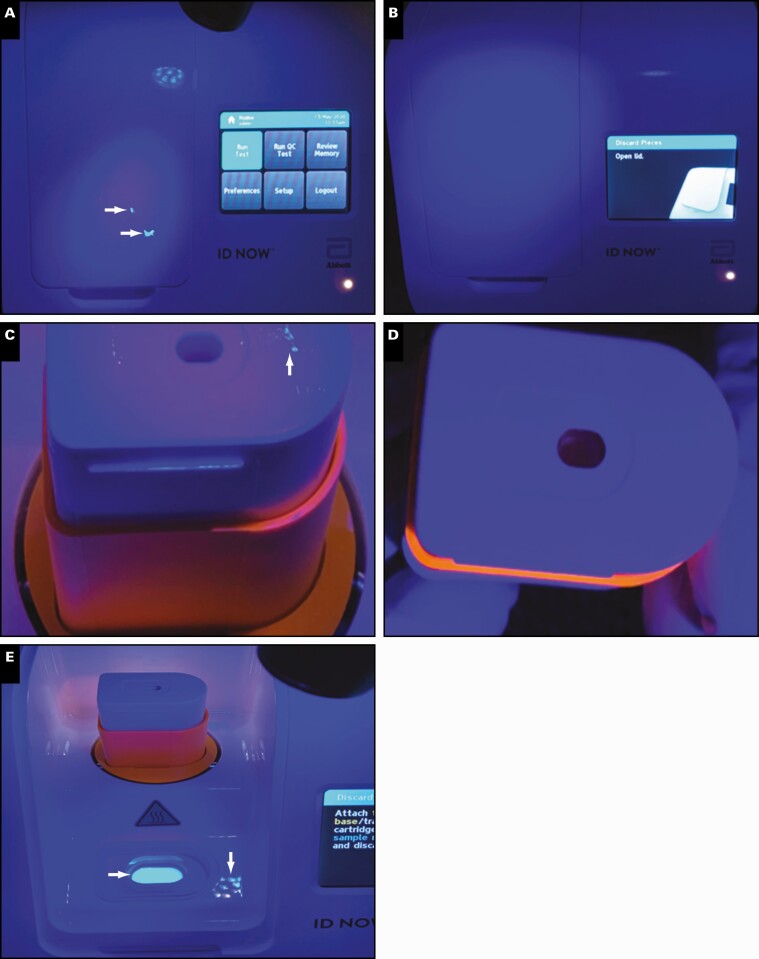
Visualization of the ID NOW and consumables following Glo Germ sample processing. **A**, Original sample handling procedures. **B**, Modified sample handling procedures. **C**, The inoculated lysis buffer cartridge. **D**, Sample transfer cartridge after modified sample handling procedures. **E**, Lysis buffer cartridge inoculated with mock Glo Germ sample.

**Figure 3:**
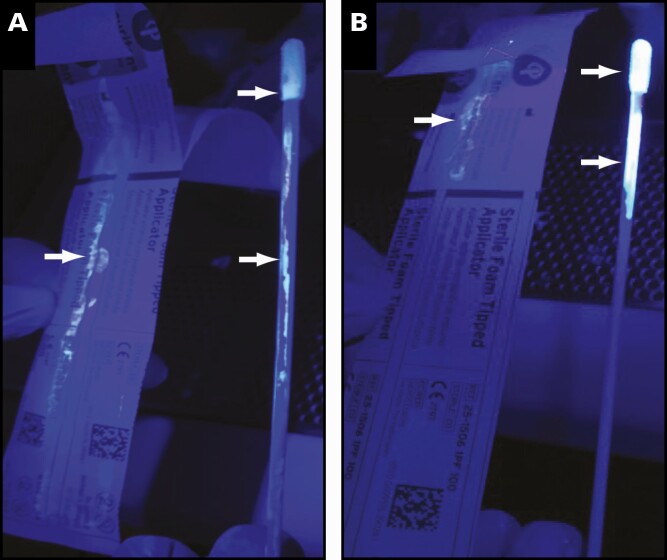
Original sample handling (**A**) and modified sample handling (**B**) showing contamination on the wrapper, stick, and tip. Contamination zones denoted by arrows.

To assess potential workflow challenges associated with the use of the ID NOW, five newly trained assay users were evaluated. As shown in **Figure 1**, users 3 and 4 had minimal traces of sample on their gloves following sample handling compared with users 1, 2, and 5, who showed traces of sample on the bottom and top of their fingertips. In addition, users 1, 2, and 5 had sample residue on the lid of the instrument and the sample transfer cartridge **Figure 2**. As expected, all users had traces of sample on the disposable swabs **Figure 3**.

As suggested by the original ID NOW instructions for use (IFU) method of transport,^12^ swabs were reinserted back into the wrapper after being used to collect patient specimens, a method that was removed from the IFU in the Emergency Use Authorization amendment.^13^ When this procedure was replicated using Glo Germ, residual sample was observed on the swab tip, stick, and wrapper **Figure 3A**. This occurs as the swab is reinserted and subsequently spreads sample along the wrapper liner. However, when modifying swab handling techniques so that swabs were only reinserted into the original wrapper partially **Figure 3B**, the spread of residual sample was minimized. As expected, after incubation of the Glo Germ mock sample in the lysis buffer cartridge, this area was illuminated with exposure to UV light (**Figure 2**).

During the workflow assessment, we observed that users had difficulty with the following: (1) attaching the ID NOW transfer cartridge to the test base (two users), (2) dispensing the sample into the test base cartridge (three users), and (3) stacking and securing the cartridges (transfer, test base, and lysis buffer) for disposal (two users). However, after gaining experience in the testing workflow, users were able to successfully conduct tests without these issues. However, if these difficulties persist, they could result in an invalid test run or potential spills during testing.

### Sofia 2 SARS Antigen FIA Workflow Assessment

**Figure 4:**
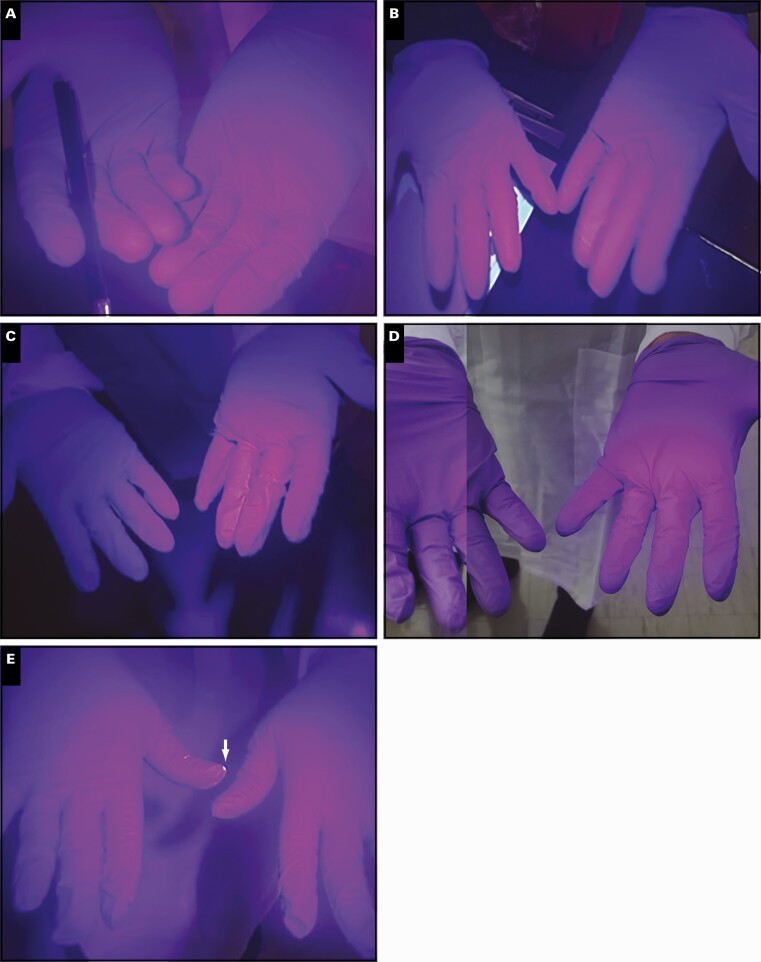
Sofia visualization of Glo Germ cross-contamination following mock sample handling. Representative of user’s gloves after processing mock Glo Germ sample on the Sofia. Contamination zone denoted by arrow. **A**, User 1, laboratory staff. **B**, User 2, nonlaboratory staff. **C**, User 3, laboratory staff. **D**, User 4, nonlaboratory staff. **E**, User 5, laboratory staff.

**Figure 5:**
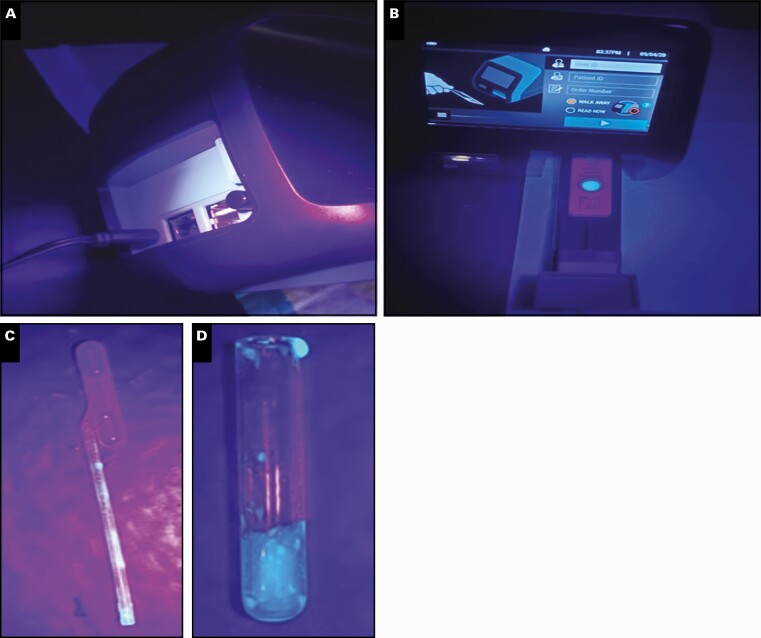
Visualization of the Sofia instrument and used kit consumables following Glo Germ sample processing. No cross-contamination of the instrument back (**A**) and front (**B**) was observed. Contamination along the reagent vial lip (**C**) and transfer pipette shaft (**D**).

Sofia workflow challenges were assessed with Glo Germ in the same manner as ID NOW. As shown in **Figure 4**, there was limited contamination on users’ fingers and the Sofia instrument. However, we observed evidence of contamination on the shaft of the transfer pipette and the upper rim of the glass reagent tube **Figure 5**.

The Sofia instrument is designed to rapidly read developed cartridges at the end of the test procedure. During the workflow assessment, we observed the following difficulties: (1) using the pipette to accurately transfer sample material from the reagent tube to the cartridge, (2) accurately dispensing the entirety of the sample into the well on the Sofia cartridge from the pipette, and (3) using the touchscreen on the instrument. We also observed that the two analysis options offered on the instrument (“run now” vs “walk away”) may cause errors in sample analysis. The “walk away” setting requires that the cartridge is inserted into the machine immediately after the sample is dispensed. The instrument times the incubation period and reads the cartridge after 15 minutes. When using the “read now” setting, the sample cartridge is incubated for 15 minutes on the benchtop and then inserted into the machine once the incubation period is complete. This allows one instrument to be used to read multiple cartridges in a 15-minute period (instead of one). However, this requires the user to manually track the incubation time for each test cartridge, which could lead to timing inconsistencies. Finally, the packaging of reagents for the Sofia may lead to potential contamination issues for minimally experienced assay users. Transfer pipettes are not individually wrapped and instead supplied in the same bags and thus could easily be contaminated when the user collects them to run each subsequent test.

### Contamination Check During Utilization Testing

The ability to detect contamination of surfaces was confirmed by simulating a spill: 100 µL each of contrived ID NOW (10× LOD) and Sofia (0.1 ng/µL) samples prepared as described above was pipetted onto a nonporous surface directly in front of the instrument. These samples were tested and found to be positive in all cases. Environmental testing by swabbing the bench was also conducted after each user’s mock run, which yielded no positive results except for one case when a spill occurred during ID NOW testing by user 3. This resulted in the positive test in environmental sample 3 **Table 6**. For the Sofia testing, all environmental samples collected tested negative for SARS-CoV-2 antigen.

**Table 6:** Swabbing of the Benchtop and Instruments to Test for Potential Contamination During Testing^a^

Sample ID	ID NOW Spill	Sofia 2 Spill
Environmental sample 0	SARS-CoV-2 negative	SARS-CoV-2 negative
Environmental sample 1	SARS-CoV-2 negative	SARS-CoV-2 negative
Environmental sample 2	SARS-CoV-2 negative	SARS-CoV-2 negative
Environmental sample 3	SARS-CoV-2 presumptive positive	SARS-CoV-2 negative
Environmental sample 4	SARS-CoV-2 negative	SARS-CoV-2 negative
Environmental sample 5	SARS-CoV-2 negative	SARS-CoV-2 negative

^a^Environmental samples from simulated spills on the ID NOW and Sofia were tested using the Cepheid Xpert Xpress SARS-CoV-2 test and Sofia SARS Antigen FIA, respectively. Environmental samples 1 to 5 were collected after completion of each test panel and correspond with user number in Table 3. A single spill during testing resulted in one presumptive positive. Mock spills were all successfully detected.

### Comparison of the ID NOW and Sofia as Viable POCT Platforms

Users with no prior laboratory experience preferred the Sofia test to the ID NOW and found Sofia instructions easier to follow. In addition, the transfer and incubation process for the Sofia was more straightforward and concise to users without laboratory experience. However, users with laboratory experience had a higher preference for the automated protocol provided by the ID NOW instrument. Experienced laboratorians also found the ID NOW system better designed and suggested that a more closed system could be less prone to spills or the transfer of inaccurate sample volumes during testing.

We compared the use of consumables and utility for both the ID NOW and the Sofia. The ID NOW used fewer open containers, and transfer of specimen was conducted using a simple cartridge system. Conversely, the Sofia required plastic pipettes for transfer of specimen and users required a rack to hold reagent vials, presenting more opportunities for work surface contamination. However, both the ID NOW and the Sofia are straightforward and easy to use and learn. Newly trained users learned proper techniques for both instruments and were able to follow directions to accurately test samples 85% of the time for the Sofia and 90% of the time for the ID NOW. Users with laboratory experience were able to accurately test samples 100% of the time.

### Development of a POCT Testing Toolkit

During our evaluation of the workflow process for both instruments, we identified procedural concerns that could affect testing. First, different testing sites may have different needs for sample processing and may make the decision to reflex samples to a reference laboratory. Samples being reflexed may need to be refrigerated, collected in different media, or collected in duplicate, so this is an important consideration. Rapid “presumptive” negative results may require additional laboratory testing to confirm results. For instance, in rapid sidewalk “outdoor” operations, exposure of rapid diagnostic tests and samples to a temperature greater than 30°C could have adverse effects on overall testing due to improper collection or storage.^10,14,15^ This decision to reflex samples needs to be carefully considered as it will affect how samples are collected and stored. Second, thorough user training is necessary for anyone operating a POCT device. Following training, a competency assessment should be administered to users to confirm that they are able to properly test samples on a given platform. This training can be supplemented using facility, instrument, and disease-specific fact sheets to help guide users. Finally, it was difficult to input patient information into these instruments; a secondary way to keep track of all patient information and the outcome of each test is needed (Supplementary Material: POC Toolkit).

## Discussion

Both the ID NOW and Sofia devices can obtain results rapidly and are relatively user-friendly systems operational by staff with minimal laboratory training and technical expertise. These assays and others were developed to rapidly detect the presence of SARS-CoV-2 in nonclinical settings.^16,17^ In this study, we aimed to simulate and examine the application of the ID NOW and Sofia devices in a nonlaboratory setting and assess usability and workflow concerns, which could aid in the rapid integration of POC testing in future disease outbreaks.^18^

To evaluate newly trained users’ ability to accurately process samples and determine if staff with limited experience can accurately process samples using the ID NOW, three spiked blinded samples were generated. Two of the five users generated false-negative results, demonstrating that false negatives during typical use could be a significant issue. There is a chance that this could be a test sensitivity issue. However, the concentration for both failed tests was two times the tested LOD, these contrived specimens were tested on the instrument prior to blinded user testing to confirm positivity, and the three remaining users, regardless of their level of laboratory experience, obtained the desired result. This suggests that the false negatives were due to user error. However, it is important to note that small sample size in this study may have led to inflated false-positive results. To further help mitigate this high false-negative rate due to operator error, we suggest that users undergo instrument-specific training followed by a competency assessment. These steps will ensure that (1) each user understands how to properly use the ID NOW instrument and (2) their ability to accurately perform the test is confirmed. For testing on the Sofia, users were provided with samples spiked with recombinant nucleocapsid protein or negative controls consisting of saline only. The rate of false negatives was lower for the Sofia, with only one user obtaining false-negative results for the same sample that was correctly identified by all other users. In this case, the false-negative results obtained are likely a result of inexperience in laboratory testing, as this user was the only one unable to detect the presence of antigen in contrived specimens on the Sofia instrument. This failure to accurately report SARS-CoV-2 results could lead to missed diagnoses and, more important, a lack of appropriate follow-up measures, including self-isolation and contact tracing.

Last, we sought to identify potential workflow challenges associated with using the ID NOW and Sofia on an open benchtop. While no SARS-CoV-2 nucleocapsid protein from the Sofia was detected in environmental swabs, we detected SARS-CoV-2 RNA from the ID NOW after a spill. This spill resulted from user error due to the cartridge locking mechanism not being fully engaged. Overall, considering that SARS-CoV-2 is relatively stable on a surface,^11,19,20^ if the surrounding workspace is not properly decontaminated, there is a potential for both secondary exposure to testing staff and contamination of subsequent specimens. A spill in a POCT setting could have significant consequences for exposure of staff and cross-contamination among specimens. While we did not observe any false positives due to spills in our study, a larger, more high-throughput site should be aware of the potential for cross-contamination, as a false-positive result can have significant implications for the patient. In addition, should these POCT instruments be used for testing during outbreaks of other pathogens, it is imperative that the pathogen-specific contamination risks be fully evaluated prior to implementation.

On the basis of our findings, we developed a toolkit for the ID NOW and the Sofia, consisting of customizable templates: (1) proposed laboratory workflows, (2) evaluation checklist, (3) instrument performance quality log, (4) patient log, and (5) secure transfer of data from the instruments to a storage device (toolkit). Before implementing either instrument into a POCT site, we suggest the following mitigation strategies: (1) the completion of site- and activity-specific risk assessments,^21^ (2) the use of a high-absorbency disposable pad changed between each specimen test to minimize workspace contamination, (3) rigorous user training and competency assessments prior to testing, and (4) strict adherence to standard hand hygiene and proper use and frequency of changing PPE precautions (ie, gloves, eye protection, laboratory gowns, and, if needed, use of a surgical mask or face shield precautions). Additional POCT guidelines and considerations have also been published by the College of American Pathologists (https://www.captodayonline.com/for-poc-molecular-pauses-plans-and-testing-precautions/) and can be considered in conjunction with our toolkit.

Although POCTs are easily deployable for field application during an emergency response, test accuracy is potentially compromised at the cost of convenience. In previous studies, the performance of POCTs has been clearly linked to the experience and training of the user, which influences the accuracy of the results.^22,23^ We observed the same for both the ID NOW and the Sofia platforms, indicating that proper training in device use, contamination prevention, and technique to ensure personnel safety is critical. Both instruments are easily deployable and provide time to results in under 20 minutes. For the Sofia, we noted that challenges could arise from the packaging of consumables and the use of the touchscreen on the device, which could lead to contamination. The touchscreen was so difficult to navigate for some users that it could lead to inaccurate recording of patient information in the instrument.

The response to the COVID-19 pandemic is constantly evolving, and so are global public health efforts to improve testing for SARS-CoV-2. POCTs can provide rapid results and allow testing to be completed in nonlaboratory settings. However, care must be taken with the use and interpretation of POCT results, as these will affect diagnosis, patient follow-up, contact tracing disease, and overall disease spread. The pros and cons of POCT implementation need to be carefully evaluated so that the spread of the pathogen being tested can be properly mitigated. In conclusion, our study highlights the importance of developing site-specific workflows and training plans and assessments for SARS-CoV-2 POCTs prior to deployment for field applications in the ongoing COVID-19 pandemic and future disease outbreaks.

## Supplementary Material

aqab081_suppl_Supplementary_Box_1

aqab081_suppl_Supplementary_Material
