# Supplementary material for: Use, Safety Assessment, and Implementation of Two Point-of-Care Tests for COVID-19 Testing
Source: Am J Clin Pathol. 2021 Jul 24:aqab081. doi: 10.1093/ajcp/aqab081 (PMC8336596; doi:10.1093/ajcp/aqab081)
Supplement: aqab081_suppl_Supplementary_Box_1 [file aqab081_suppl_supplementary_box_1.docx]

**Supplementary information**

**Supplementary Box 1.** Interview questions asked of each of the users that conducted testing with blinded and Glo Germ samples to determine the instrument with the optimal usability.

| **ID NOW vs Sofia Interview Template**   1. Were the instructions for both the ID NOW and Sofia clear? Was there anything you did not understand or that confused you? 2. Were there any steps of testing with the ID NOW or the Sofia that you found particularly difficult? 3. Were there any steps with either instrument that you found particularly well designed? 4. After conducting testing with both the ID NOW and the Sofia, which do you find to be more user friendly?    1. Why do you think this?    2. What attributes of your preferred device work the best for you? 5. Do you think that the ID NOW or the Sofia would be easier to deploy in non-clinical settings?    1. Why do you think this?    2. Is there anything that you think would make either of these instruments easier to deploy in a non-clinical setting? 6. Which instrument protocol do you think you would have an easier time teaching to a new user, and why do you think that is? |
| --- |
