# Supplementary material for: Use, Safety Assessment, and Implementation of Two Point-of-Care Tests for COVID-19 Testing
Source: Am J Clin Pathol. 2021 Jul 24:aqab081. doi: 10.1093/ajcp/aqab081 (PMC8336596; doi:10.1093/ajcp/aqab081)
Supplement: aqab081_suppl_Supplementary_Material [file aqab081_suppl_supplementary_material.docx]

**Point-of-Care (POC) Instrument Testing Toolkit**

A guide for implementation

of POC instruments into your workflow.

Compiled November 2020

**Table of Contents**

1. Sample collection and reflexing to a reference laboratory P.3
   1. Determine specimen type
   2. Determine required storage conditions
   3. Considerations for reflexing specimens
   4. Plan for specimen collection
      1. Decision tree
2. User training and competency assessment P.5
   1. Sample user competency assessment
   2. Abbott ID Now Quick Facts Sheet
   3. Sofia SARS Antigen FIA Quick Facts Sheet
3. Quality assurance and documentation P. 11
   1. Fact Sheet for patients: COVID-19 example
   2. Failed External QC Log
   3. Patient QC Log
4. Patient tracking P. 16
   1. Sample patient tracking log
5. **Sample collection and reflexing to a reference laboratory**

- The type of specimen collected needs to correspond with what the Point-of-Care (POC) instrument manufacturer requires. For COVID-19 testing, the most common specimen types are:
  - Nasopharyngeal swabs
  - Nasal or nares swabs
  - Oropharyngeal swabs
- Once the specimen is collected, it must be properly stored until tested. Consider the following when storing your specimens:
  - At what temperature does your POC Instructions for Use (IFU) require specimens be stored?
  - What transport media, if any, for specimen storage is compatible with your device?
- Facilities must follow local, state, and CDC guidance for reflexing specimens to a reference laboratory for further testing. Some examples as to why samples may be reflexed are below.
  - Positive specimens may be reflexed for:
    - Testing using a more specific assay to rule out the possibility of false positives
    - Further analysis such as whole genome sequencing
    - Storage
  - Negative specimens may be reflexed for:
    - Testing using a more sensitive assay to rule out false negatives
    - Storage
- Once the decision to reflex is made, your testing center can use the following decision tree to make sure samples are being collected properly for your desired outcome.

1. **User training competency assessment**

| **Trainee:** | **Date of Training:** |
| --- | --- |
| **Test Name:** | **Trainer:** |

This document is used to verify that the trainee has reviewed provided materials and received sufficient training for running COVID-19 tests at __________________________ facility. Training to include the following:

- Review of the test protocol.
- Review of the SARS CoV-2 CDC Guidelines for point-of-care testing. (<https://www.cdc.gov/coronavirus/2019-ncov/lab/point-of-care-testing.html>)
- Observed in understanding analyzer components, cleaning, and QC requirements.
- Successful performance of the COVID-19 test on contrived specimens, interpretation of results, and reporting of results.

| Test protocol for review can be found: | Date of review: |
| --- | --- |

**Observation of the testing process completed by the trainee**

| **Steps** | **Observation notes and pass or fail** | **Trainer’s initials** | **Date Complete** |
| --- | --- | --- | --- |
| **Donning PPE**   - Wash hands - Don gloves of proper size - Don laboratory gown (disposable preferred) - Don face mask/shield (if recommended) | □ PASS □ FAIL |  |  |
| **Decontamination of testing area** | □ PASS □ FAIL |  |  |
| **Confirm necessary components of kit are present**   - Reagents - Consumables - QC materials - Quick reference protocol - Expiration date   *This section can be revised to reflect specific components of POCT device being used. | □ PASS □ FAIL |  |  |
| **Observes proper storage of reagents:**   - Correct temperature for instrument - Correct temperature for reagents | □ PASS □ FAIL |  |  |
| **Operate the analyzer/instrument including turning off, turning on, and re-boot protocols** | □ PASS □ FAIL |  |  |
| **Understands the importance of stringent cleaning and changing of PPE between each patient test**   - Uses 70% ethanol or freshly prepared bleach solution - Allows surfaces to dry - Discard all cleaning materials in red biohazardous waste container with closing lid | □ PASS □ FAIL |  |  |
| **Testing procedure**   - Checks patient medical records for completeness - Enters patient medical ID number into the instrument or the patient tracking spreadsheet - Allows reagents to come to room temperature, *if necessary* - Conducts processing of specimen according to manufacturer’s or facility protocols - Records the patient result - Reports the patient result - Properly stores remaining specimen (if any) - Decontaminates the testing area - Changes PPE (if necessary) | □ PASS □ FAIL |  |  |
| **Running quality control**   - Understands the importance of keeping track of each new shipment of reagents or testing instrument - Runs QC testing according to manufacturer’s instructions - Is aware of necessary frequency of QC testing - Is aware of events that require QC to be completed (such as moving the instrument) | □ PASS □ FAIL |  |  |
| **Results interpretation and reporting**   - Understands that tests may have positive, negative, or invalid results, and how to distinguish between these outcomes - Understands it is necessary to repeat tests when an invalid result is obtained and what protocol to follow should repeat invalid results be obtained - Treats a positive result as an alert and follows institution policy for instrument decontamination and replacement of PPE - Documented appropriate patient test result in patient tracking document | □ PASS □ FAIL |  |  |
| **Proficiency testing**   - Reads and follows POC kit instructions - Knows not to share or discuss results outside of reporting guidelines - Reports back the results within the expected time period for the laboratory | □ PASS □ FAIL |  |  |
| **Limitations of test**   - Understands that false negatives can occur due to poor specimen collection or handling and if the POC protocol is not followed - Understands that most POC tests cannot rule out the presence of other viral or bacterial pathogens | □ PASS □ FAIL |  |  |
| **Troubleshooting**   - Does the user understand common errors associated with the instrument and why they occur? - Can the user assess an issue during testing, and respond properly? - Does the user know the proper technical service number or email should contacting them be necessary? - Does the user understand the proper chain of command for seeking assistance or reporting errors? | □ PASS □ FAIL |  |  |

**Additional Notes**:_______________________________________________________________ __________________________________________________________________________________________________________________________________________________________________________________________________________________________________________

I have received orientation and training on the above specified test and I have the knowledge to perform basic tasks required.

______________________________________________________ ____________

Trainee Signature Date

I have observed the trainee and have confidence that they can complete the above tasks with minimal supervision.

______________________________________________________ ____________

Trainer Signature Date

1. **Quality Assurance documentation**

**Abbott ID Now Quick Facts Sheet**

**Authoriozation for use of a test in the CLIA waived setting, which is determined by Centers for Medicare (CMS) and allows the test to be used outside clinical laboratory settings, indicates the following:**

- - A facility MUST have at least a Clinical Laboratory Improvement Amendments (CLIA) Certificate of Waiver to operate. Please also note that certain states may require different certificates or permits.
  - A laboratory specific validation or verification of the assay (this does not refer to QC) does not need to be performed
  - Negative results should be treated as presumptive. Refer to Instructions for Use (IFU): <https://www.fda.gov/media/136525/download>
- Before operating the Abbott ID NOW
  - Review biosafety recommendations and conduct site-specific biosafety risk assessment
  - Please refer to the manufacturer’s IFU and complete training for all operators

***Precaution: Reagent kit components are not interchangeable with components from kits designed for other assays.**

- An instrument cannot be used to test patient specimens until positive and negative quality control (QC) samples (provided by manufacturer) have been successfully run and the results documented.
  - Manufacturer “internal” QC refers to the procedural “Run QC Test” instructions on the ID NOW Instrument during the instrument initialization step. Please refer to instrument user manual for further details.
  - It is recommended to maintain a quality control log and patient and/or specimen log
  - Please refer to customizable QC – patient log worksheet template
- External QC MUST be run prior to use of each reagent kit shipment and/or lot
  - External QC refers to the “Positive and Negative Control Swabs” contained within each ID NOW COVID-19 test Kit.
  - It is recommended that QC be performed once a day before operational use of instrument.
  - Instructions for Use (IFU): <https://www.fda.gov/media/136525/download>
  - Recommended to run External QC on each device. Note, the external QC is for the reagent kits. The device has an internal QC system.
  - In the event of an external QC failure, complete “Failed External QC Reporting Log” and email to [ts.scr@abbott.com](mailto:ts.scr@abbott.com).
  - Training: Prior to operational use, each operator should perform one run using at least one of the QC reagents (Positive or Negative QC) This can be done at the same time as running QC for reagent kit shipment
- Safety and operation guide:
  - Perform site-specific biosafety risk assessment before testing.
  - Use appropriate personal protective equipment (PPE) - gloves, lab coats/gowns, face shield or eye protection and face mask, for splash and splatter precautions
  - Operate the instrument within a biosafety cabinet, *whenever possible*
    - If a biosafety cabinet is not available, then place instrument away from where others are working and have staff operating the instrument wear PPE (gloves, lab coats/gowns, face shield or eye protection and face mask)
    - Consider placing a sheet of plexiglass in front of the instrument to further guard against splashes
  - Biosafety Practices for COVID-19 Testing Using Abbott ID Now
    - FDA Fact Sheet <https://www.fda.gov/media/136523/download>
    - CDC Updated POC guidance: <https://www.cdc.gov/coronavirus/2019-nCoV/lab/lab-biosafety-guidelines.html>
    - Abbott has Webex training available by appointment

**Sofia SARS Antigen FIA Quick Facts Sheet**

- **CLIA waived status, which is determined by CMS and allows the test to be used outside clinical laboratory settings, indicates the following:**
  - A facility MUST have at least a Clinical Laboratory Improvement Amendments (CLIA) Certificate of Waiver to operate.
  - A laboratory specific validation or verification of the assay (this does not refer to QC) does not need to be performed
  - Negative results should be treated as presumptive. Refer to Instructions for Use (IFU): <https://www.fda.gov/media/137885/download>
- Before operating the Sofia SARS Antigen FIA
  - Please refer to the manufacture’s packaging instructions and complete training for all operators.
  - Review biosafety recommendations and conduct site-specific biosafety risk assessment.
- ***Precaution: Reagent kit components are not interchangeable with components from kits designed for other assays.**
- During the initial setup, each device cannot be operated until a positive and a negative QC swab has been run
  - It is recommended to maintain a quality control log and patient and/or specimen log
  - Please refer to customizable QC – patient log worksheet template
- External QC MUST be run prior to use of each reagent kit shipment and/or lot
  - It is recommended that QC be performed once for each untrained operator, once for each new shipment of kits, and as deemed additionally necessary by your internal quality control procedures and in accordance with Local, State, and Federal regulations or accreditation requirements.
  - Instructions for Use (IFU): <https://www.fda.gov/media/137885/download>
  - In the event of an external QC failure, complete “Failed External QC Reporting Log” and email to [technicalsupport@quidel.com](mailto:technicalsupport@quidel.com).
  - Training: Prior to operational use, each operator should perform one run using at least one of the QC reagents (Positive or Negative QC) under the supervision of a trained laboratorian. This can be done at the same time as running QC for reagent kit shipment.
- Safety and operation guide:
  - Perform site-specific biosafety risk assessment before testing
  - Use appropriate personal protective equipment (PPE) - gloves, lab coats/gowns, face shield or eye protection + face mask, for splash and splatter precautions
  - Operate the instrument within a biosafety cabinet, *whenever possible*
    - If a biosafety cabinet is not available, then place instrument away from where others are working and have staff operating the instrument wear PPE (gloves, lab coats/gowns, face shield or eye protection + face mask)
    - Consider placing a sheet of plexiglass in front of the machine to further guard against splashes.
  - Biosafety Practices for COVID-19 Testing Using Sofia SARS Antigen FIA
    - FDA Fact Sheet <https://www.fda.gov/media/136523/download>
    - CDC Updated POC guidance: <https://www.cdc.gov/coronavirus/2019-nCoV/lab/lab-biosafety-guidelines.html>

**Failed External QC Reporting Log**

**Location/testing site**: ____________________________________________________

**Date tested**: _____________________

**Test kit Lot**#_______________________ **Test Kit Lot expiration date**: __________________

1. **Negative QC Failed**? Yes or No (please circle one)

If Yes, please provide the lot number and expiration date of the failed control:

**Lot#**____________

**Expiration date**: _______________

**Indicate corrective action performed**: ____________________________________

____________________________________________________________________________________________________________________________________________________________________________________________________________________________________________________________________________________

1. **Positive QC failed**? Yes or No (please circle one).

If Yes, please provide the lot number and expiration date of the failed control:

**Lot#**____________

**Expiration date**: _______________

**Indicate corrective action performed**: ____________________________________

____________________________________________________________________________________________________________________________________________________________________________________________________________________________________________________________________________________

**Review by**: _______________________________________ **Date**:___________________

Please send this form to:__________________________________________________________

**Patient/QC log Sheet**

**Month/Year:__________________________________ Kit Lot#/Exp Date:_____________________**_____________

**POS QC Lot#/Exp Date:_________________________ NEG QC Lot#/Exp Date:_________________________**

****Ensure that external QC is performed once each day before beginning patient testing and with each new lot/shipment.**

| **Date** | **Patient ID or Label** | **Patient Result**  **Neg / Pos / Invalid**  ***If Invalid, repeat test** | **External POS QC** | **External NEG QC** | **Tech** | **Reviewed By** |
| --- | --- | --- | --- | --- | --- | --- |
|  |  |  |  |  |  |  |
|  |  |  |  |  |  |  |
|  |  |  |  |  |  |  |
|  |  |  |  |  |  |  |
|  |  |  |  |  |  |  |
|  |  |  |  |  |  |  |
|  |  |  |  |  |  |  |
|  |  |  |  |  |  |  |

**Reviewed by**:_____________________________________________

IV. Patient Tracking

**Patient Results Tracking Log**

| **Date** | **Patient/Sample ID**  **(or label)** | **Patient Result**  **Please check (✓) to indicate test result** | **Operator’s initials** | **Entered in patient chart and verified**  **✓=Task completed** | **Reviewer’s initials and date** | **Result reported to health department**  **✓=Task completed** |
| --- | --- | --- | --- | --- | --- | --- |
|  |  | □ NEGATIVE  □ POSITIVE  □ INVALID/TEST REPEATED |  |  |  |  |
|  |  | □ NEGATIVE  □ POSITIVE  □ INVALID/TEST REPEATED |  |  |  |  |
|  |  | □ NEGATIVE  □ POSITIVE  □ INVALID/TEST REPEATED |  |  |  |  |
|  |  | □ NEGATIVE  □ POSITIVE  □ INVALID/TEST REPEATED |  |  |  |  |
|  |  | □ NEGATIVE  □ POSITIVE  □ INVALID/TEST REPEATED |  |  |  |  |
|  |  | □ NEGATIVE  □ POSITIVE  □ INVALID/TEST REPEATED |  |  |  |  |
